# An Unusual Case of Candida kefyr Fungemia in an Immunocompromised Patient

**DOI:** 10.7759/cureus.14138

**Published:** 2021-03-27

**Authors:** Lakshmi Jyothi, Naveen P Reddy, Shazia Naaz

**Affiliations:** 1 Microbiology, All India Institute of Medical Sciences, Hyderabad, IND; 2 General Medicine, Mediciti Institute of Medical Sciences, Hyderabad, IND; 3 Microbiology, Employees' State Insurance Corporation (ESIC) Medical College & Hospital, Sanathnagar, Hyderabad, IND

**Keywords:** candida kefyr, plasmapheresis, guillain barre syndrome

## Abstract

An immunocompromised patient with a known history of cerebrovascular accident (CVA) with right-sided hemiparesis of long duration, type 2 diabetes mellitus, and hypertension presented with signs of rhabdomyolysis and later acute kidney injury (AKI). He subsequently developed Guillain Barre syndrome. Initially, hemodialysis was followed by plasmapheresis. After hemodialysis, the patient presented with multiple episodes of vomiting and weakness of all limbs. A culture showed growth of *Enterococcus faecalis,* and on Day 6, a bloodstream infection with *Candida kefyr* and a urinary tract infection with *Enterococcus faecalis *were diagnosed. We report a rare case of bloodstream infection due to *C. kefyr*.

## Introduction

*Candida kefyr* (previously known as *Candida pseudotropicalis*) is a yeast with its teleomorph currently recognized as *Kluyveromyces marxianus*. *C. kefyr* is an emerging infectious agent in cases with oncohematological malignancies. It has been frequently isolated from oncohematology wards more often than other wards [1]. These species are susceptible to azoles, echinocandins, amphotericin B, and flucytosine. The reason for the preponderance of this pathogen in the immunocompromised host is still unknown [1-2]. This case involved a patient with statin-induced rhabdomyolysis and Guillain Barre syndrome (GBS). The patient was later diagnosed with *C. kefyr* fungemia. Treatment with intravenous immunoglobulin or plasma exchange and antifungal drugs was carried out, and the patient recovered completely.

## Case presentation

A 51-year-old male patient with a history of diabetes, hypertension, diabetic retinopathy, and a known case of cerebrovascular accident (CVA) with right-sided hemiparesis was initially admitted because of complaints of polyarthralgia (multiple episodes) with rhabdomyolysis and acute kidney injury (AKI).

Two weeks after the initial admission, he was readmitted with symptoms of Guillain Barre syndrome. Laboratory investigations showed urine albumin levels (24 hours) = 38.5 mg/dL (normal range: (24 hours) < 30 mg/dL), creatine phosphokinase (CPK) = 410 IU/L (normal range: 39 - 308 IU/L), aspartate aminotransferase (AST) = 54 IU/L (normal range: 5 - 40 IU/L), alanine aminotransferase (ALT) = 59 IU/L (normal range: 5 - 40 IU/L), lactate dehydrogenase (LDH) = 320 U/L (normal range: 135 - 225 U/L), and serum potassium levels = 5.9 mmol/L (normal range: 3.6 - 5.2 (mmol/L).

**Figure 1:**
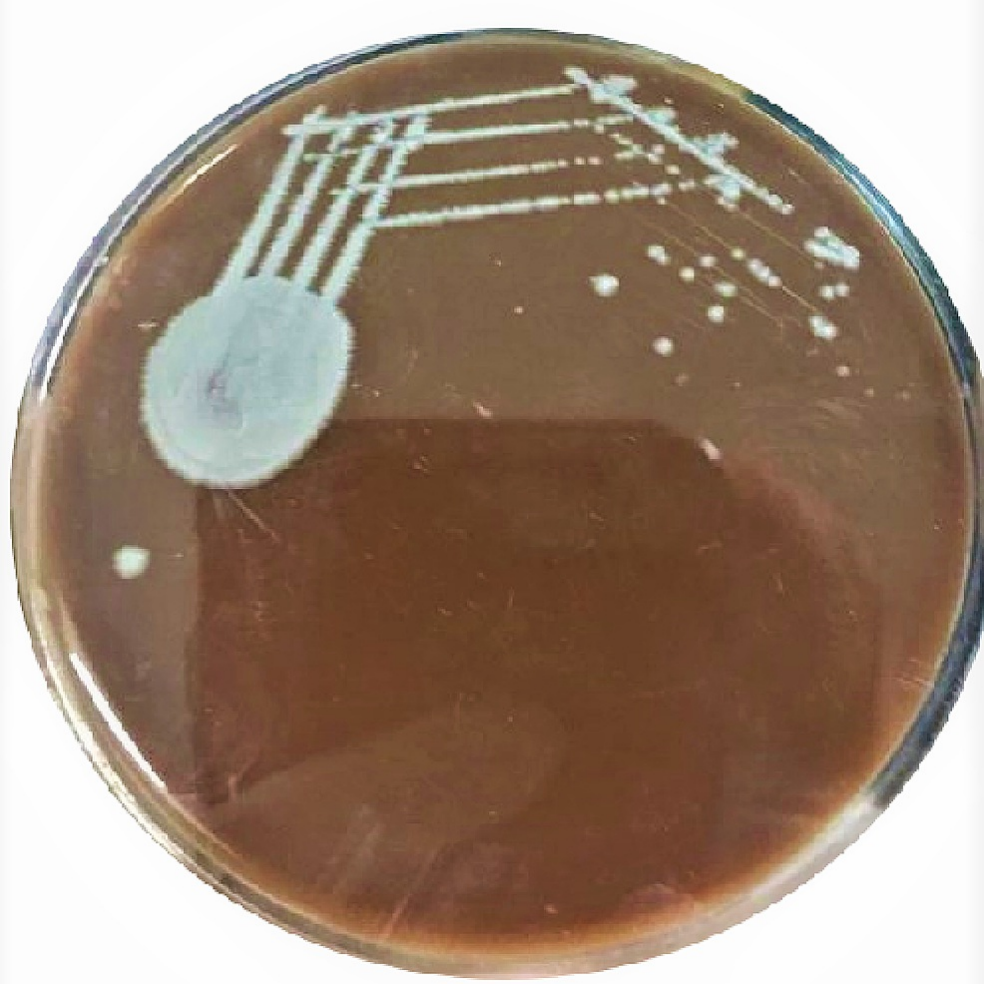
Candida kefyr colonies on blood agar

**Figure 2:**
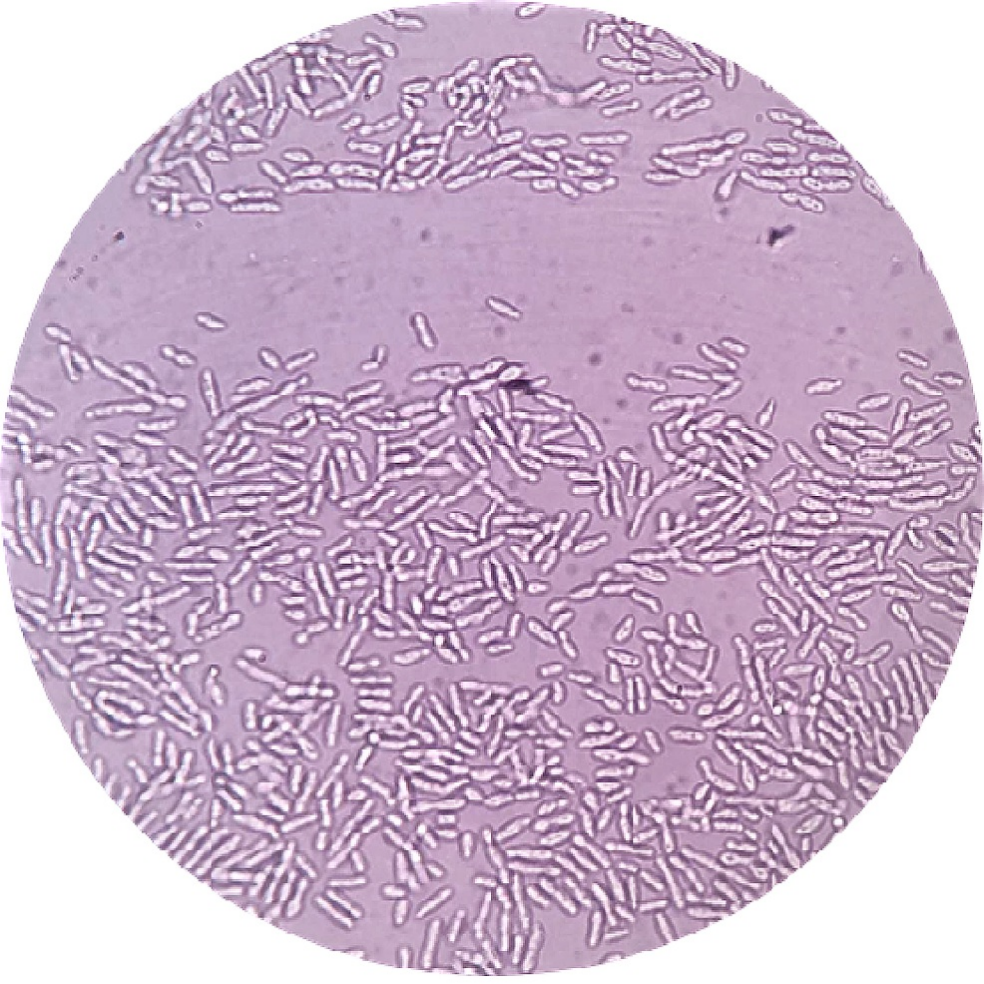
Potassium hydroxide (KOH) wet mount showing Candida kefyr colonies 40X

On examination, the patient was on a Foley catheter and presented with oliguria. Blood pressure was 140/90 mmHg and his temperature was 98.6ºF. Dialysis was recommended, and a right-sided femoral cannula was inserted. He later developed Guillain Barre syndrome. Initially, hemodialysis was followed by plasmapheresis. After hemodialysis, the patient presented with multiple episodes of vomiting and weakness of all limbs. The patient received eight units of fresh frozen plasma (FFP), intravenous (IV) Ringer's lactate 500 ml given in dialysis, IV normal saline 500 ml, IV calcium gluconate 10 ml, 5% albumin, and heparin 5,000 IU. There was a rise in the serum creatinine and potassium levels, and the patient was transferred to acute neuro care after 24 hours. Urine and blood samples were sent for culture and sensitivity. The culture showed growth of *Enterococcus faecalis,* and on Day 6, a bloodstream infection with *C. kefyr* and urinary tract infection with *E. faecalis *was diagnosed. Processing was done according to the standard microbiological procedure in the aerobic blood culture bottle and incubated in the Bactec 9050 Blood Culture System (Becton Dickinson India Pvt. Ltd., Gurgaon Haryana, India). After 72 hours, a subculture on Sabouraud dextrose agar, blood agar was carried out. Colonies on blood agar and Sabouraud dextrose agar were opaque, smooth white colonies (Figure 1). A 10% potassium hydroxide (KOH) mount showed abundant production of pseudohyphae. A streak of young actively growing yeast was made down the center of the agar, followed by three to four streaks across the first to dilute the inoculum (Figure 2). This is was covered with a coverslip and incubated for 72 hours at room temperature. The plate was examined using 10X and 40X elongated blastoconidia were seen arranged parallelly, like the typical “logs in a stream” appearance. The Candida species were identified using Rapid Yeast ID with MicroScan WalkAway 96S (Beckman Coulter, Brea, CA, USA) which showed 99.99% as *C. kefyr*. Susceptibility testing was done using E-test strips on antifungal assay agar. The isolate was found sensitive to fluconazole, itraconazole, anidulafungin, voriconazole, and amphotericin B. Fluconazole, 400 mg/day, was started and continued for a period of three months. The patient recovered and was discharged successfully with continuous follow-up for the next six months.

## Discussion

*C. kefyr* is considered an opportunistic pathogen in the case of a breakdown of the body's immune system. *C. kefyr* is an emerging pathogen among patients with hematologic malignancies. Dufresne et al. studied *C. kefyr* colonization and infection in hematological malignancy patients over a period of six years and found that 83 patients were colonized and/or infected with *C. kefyr*, with eight (9.6%) having invasive candidiasis [3].^ ^Sendid et al. reported a two-fold detection rate of *C. kefyr* isolates from adult patients in oncohematology wards compared to patients in other wards [1]. Isolation of *Candida* in blood cultures should not be taken as a contaminant and efforts should be directed to search for the source of the bloodstream infection [4]. In this case report, the multiple sittings of hemodialysis with a background of diabetes mellitus type 2 and ongoing treatment with immunomodulators could have led to the infection with *C. kefyr*. Higher doses of fluconazole, 400 mg/day, were given for 75 days after stem cell transplantation and were reported to provide clinically important protection against invasive yeast infection [5]. Because prophylactic fluconazole treatment (100 mg and then 200 mg/day, orally) in stem cell transplant recipients has been associated with the emergence of infection due to fluconazole-resistant, non-albicans species of *Candida*, patients often do not receive fluconazole prophylaxis. However, this case was successfully treated with prolonged treatment for three months with fluconazole, 400 mg/day.

## Conclusions

Emerging non-albicans *Candida *species are becoming important causes of nosocomial infections. *C. kefyr *is one such organism. Early clinical suspicion with a prompt diagnosis and treatment is of high importance in such cases in order to decrease the morbidity and mortality.
